# Retinal tissue preparation for high-resolution live imaging of photoreceptors expressing multiple transgenes

**DOI:** 10.1016/j.mex.2018.03.001

**Published:** 2018-03-16

**Authors:** Mohammad Haeri, Xinming Zhuo, Morteza Haeri, Barry E. Knox

**Affiliations:** a Departments of Neuroscience & Physiology, and Ophthalmology, SUNY Upstate Medical University, Syracuse, NY, United States; b Department of Pathology & Immunology, Baylor College of Medicine, Houston, TX, United States; c Department of Molecular & Human Genetics, Baylor College of Medicine, Houston, TX, United States; d Department of Chemical & Biomedical Engineering, Syracuse University, Syracuse, NY, United States; e Department of Pathology & Laboratory Medicine, David Geffen School of Medicine at UCLA, Los Angeles, California

**Keywords:** Retinal tissue preparation for high-resolution live imaging, Live imaging, Retinal, Photoreceptor, *Xenopus*

## Abstract

Live imaging has become the favorite method in recent years to study the protein transport, localization and dynamics in live cells. Protein transport is extremely essential for proper function of photoreceptors. Aberration in the proper transport of proteins gives rise to the loss of photoreceptor and blindness. On the other hand, the ease of generation of transgenic *Xenopus laevis* tadpoles and the advantage of high resolution live confocal imaging provide new insight into understanding protein dynamics in photoreceptors. There are several steps for quantifying and visualizing fluorescently tagged proteins in photoreceptors starting with assembly of plasmids, generation of transgenic tadpoles, preparation of retinal tissues, imaging the transgenic photoreceptors and finally analyzing the recorded data. The focus of this manuscript is to describe how to prepare retinal tissues suited for live cell imaging and provide our readers with a tutorial video. We also give a summary of steps leading to a successful experiment that might be designed for imaging the ultrastructures of photoreceptors, the expression of two or more different fluorescently tagged proteins, their localization, distribution, or protein dynamics within photoreceptors.

• Retinal tissue live imaging demonstrates the ultrastructures of photoreceptors.
• High resolution live confocal imaging provides new insight into understanding the pathophysiology of photoreceptors.

## Graphical abstract

**Figure:**
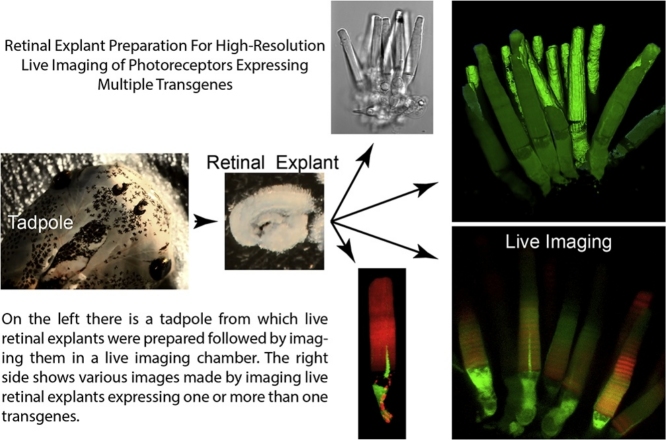

## Specifications table

**Table:** 

Subject area	• *Neuroscience*
More specific subject area	*Retinal*
Method name	*Retinal Tissue Preparation For High-Resolution Live Imaging*
Name and reference of original method	*Haeri, M. & Knox, B. E. Rhodopsin mutant P23H destabilizes rod photoreceptor disk membranes. PLoS One 7, e30101, doi:10.1371/journal.pone.0030101 PONE-d-11-21552 [pii] (2012).*
Resource availability	

## Method details

### Introduction

Large photoreceptors in *Xenopus laevis* are well suited for live imaging and study of fluorescently tagged proteins made in the retinal cells of transgenic species. The generation of transgenic *Xenopus laevis* tadpoles expressing fusion or tagged proteins is easy, cost-effective and fast. Hundreds of transgenic frogs expressing transgenic proteins in rod photoreceptors have been made after our first report in which the expression of eGFP in rod photoreceptors using rhodopsin promoter was demonstrated [1]. Several labs adopted this method to express tagged or untagged proteins in the rod photoreceptors of *Xenopus laevis* [[2], [3], [4], [5], [6], [7], [8], [9], [10]]. Here we demonstrate how live imaging of fluorescently tagged proteins in transgenic tadpoles can be utilized to study the protein transport and dynamics in photoreceptors. In addition to recording high resolution images from live photoreceptors, we managed to utilize other imaging techniques such as fluorescence recovery after photobleaching (FRAP) or fluorescence resonance energy transfer (FRET) to demonstrate protein dynamics in photoreceptor disk membranes [11]. Moreover, we studied the fragility of freshly isolated live rod photoreceptors and discussed how pathological alterations of disk membranes by mutant proteins may lead to an increased outer segment (OS) fragility and breakage [8,12].

The structure of rod photoreceptor is unique compared to other cell types and essential for its proper function. Live imaging of photoreceptors is an opportunity to probe various hypotheses explaining the unique structures seen in these specialized cells [13]. Live imaging of cells has been described previously with descriptive methodology; however, this is not an easy task to be described solely by words. The aim of the current manuscript is not to explain how to make transgenic tadpoles as it has been explained previously in great detail [14]. Moreover, details of how to utilize live imaging for quantitative studies and image analysis of recorded data have been published by several other investigators [6]. Our goal in this report is to show how to prepare retinal tissues for live imaging and to give a summary of how different types of studies can be done using live imaging (see the Supplemented video).

## Materials and methods

### Solutions

MMR (Marc’s modified ringer, final concentration of 1× MMR in mM: NaCl, 1000; KCl, 20; MgSO_4_, 10; CaCl_2_ dihydrate, 20; HEPES, 50 and the pH = 7.4).

Ringer solution (1× Ringer solution in mM: NaCl 111, KCl 2, CaCl_2_ 1, MgCl_2_ 1, MgSO_4_ 0.5, NaH_2_PO_4_ 0.5, HEPES 3, glucose 10, EDTA 0.01).

1. *Transgenic animals and in vitros:*

1.1 Transgenic tadpoles were produced, according to the standard procedure to incorporate the desired gene into the genome of *Xenopus* sperm, using restriction enzyme-mediated integration (REMI) [1] with some modifications [[14], [15], [16]]. In this case, we used XOP-eGFP (*Xenopus* opsin promoter driving the expression of eGFP) and XOP-mCherry linearized plasmids among others including XOP-Rho-eGFP (*Xenopus* opsin promoter driving the expression of eGFP tagged opsin), XOP-Rho-eGFP(P23H) (*Xenopus* opsin promoter driving the expression of eGFP tagged mutant opsin (P23H), XOP-Rho-mCherry (*Xenopus* opsin promoter driving the expression of mCherry tagged opsin), XOP-Arrestin-eGFP (*Xenopus* opsin promoter driving the expression of eGFP tagged arrestin) and XOP-eGFP-dGryGry (*Xenopus* opsin promoter driving the expression of double geranylated eGFP) [11,17].

1.2 Phenotypically normal embryos [14] were sorted and transferred into 0.1× MMR for 6 days at 16 °C and 12/12-light/dark controlled incubator. The embryos were transferred into fresh 0.1× MMR every day. (The low temperature is optimal for lowering the rate of bacterial growth and suitable for development of embryos.)

1.3 To screen for transgenic tadpoles, the eye fluorescence of tadpoles regardless of expressing a soluble or fusion protein was examined using a fluorescence-dissecting microscope (Zeiss) (10× magnification, FITC filter for eGFP [excitation BP 450–490 nm, emission; BP 505–530] and for mCherry [excitation BP 530–585 nm, emission; LP 615 nm) on day 6–7 after fertilization (dpf) and repeated on day 20 by. Accordingly, 30 μl of 10% Tricaine (3-amino benzoic acidethylester) was added to each plastic plate (with approximately 50 ml of 0.1× MMR) containing swimming tadpoles. After 10 min the tadpoles were motionless and lay on their sides while their eyes were deviated upward at a suitable position to shed the light into their eyes. We did not keep the tadpoles in Tricaine more than 30 min.

2. *Detection of transgenic species:*

2.1 All tadpoles were scored for the intensity and uniformity of the fluorescence at day 20 post-injection (3+ for bright and uniform, 1+ for dim and/or punctate and 2+ for anything between 3+ and 1+).

2.2 The tadpoles were washed several times in 0.1× MMR until they started swimming and then were maintained in 0.1× MMR.

3. *Transgenic tadpoles and natural light rearing* [14]*:*

3.1 Transgenic *Xenopus laevis* tadpoles were kept in a room with natural light cycling to mimic their native equatorial habitat and at pH 7.8, 20 °C. Accordingly, fish tanks were exposed to natural light (13 h light and 11 h dark).

3.2 Tadpoles were fed once a day and kept in a controlled light/temperature condition for four weeks before the experiment. All animal handling and experiments were in agreement with the animal care and use guidelines at Association for Research in Vision and Ophthalmology (ARVO). All steps were performed in a similar way for all animals.

4. *Euthanization*: The euthanization and tissue processing of animals were performed in dark or minimal illumination.

Frogs/tadpoles were transferred into 10% Tricaine for 10–15 min before decapitation according to Institutional Animal Care and Use Committee (IACUC) guideline. The euthanized animal was washed quickly with water, decapitated and pithed.

5. *Preparation of retinal tissue:*

5.1 In tadpoles the eye is removed quickly and placed into a drop (50 μl) of frog ringer solution. Using a pair of tweezers, the retina is separated from the RPE (Supplemental video).

5.2 In frogs, the head was placed under a dissecting microscope followed by removal of the skin and cutting the tissue and extra-ocular muscles around the eye globe to release the whole eye. It is critical to use curved scissor to prevent laceration of the eye.

5.3 The eye globe was washed with 1× Ringer solution and immersed into a drop of 1× Ringer while the cornea is facing upward.

5.4 A hole was made into the central cornea by a sharp needle, and using a tiny sharp pair of scissors the cornea was cut all around the corneal limbus (the border of the cornea and the sclera).

5.5 A pair of tweezers was used to take out the lens without damaging the retina. The sclera was cut from one side toward the optic nerve all the way around to the other side and was removed from the eye. While holding to the edge of retina with a tweezers, the RPE was peeled off from the retina.

(The preparation of retinal tissues from tadpoles and frogs are similar but it is executed on a much smaller eye for tadpoles.)

5.6 The cleaned retina was placed in a fresh drop of ringer and was minced into pieces using a sharp razor (watch the video for a better visualization).

Note: Some investigators might need dark adapted retina for their experiments. In this case, frogs will be dark adapted in a light-sealed chamber for at least 6 h before the experiment (or according to the protocol), and all steps will be carried out in dark. Accordingly, the dissecting microscope needs to be equipped with an infrared viewer (mounted on the eyepiece of the microscope) such as, FIND-R-SCOPE Infrared Microscopy System or similar devices.

6. *Imaging chamber and live cell imaging:*

6.1 Prepare imaging chambers. The imaging chamber was made in the center of a 5 cm plastic Petri dish as described previously [6,8,9] by creating a 6-mm hole by a press drill; a number 1 coverslip covered the bottom of the chamber for the access of the microscope lenses. To glue the coverslip, wax or silicon can be used. Alternatively, commercial glass bottom plate with larger central hole are available that can be used as imaging chambers.

6.2 The retinal tissues were transferred using a cut pipette tip into imaging chamber already half filled with Ringer. The chamber was placed onto the microscope stage for imaging. All imaging was performed at 20 °C. (The low temperature is especially important in reducing the noise associated with thermal activation of rhodopsin in electrophysiological studies [18].)

7. *Live confocal microscopy*:

The recording chamber was initially searched for retinal tissues with parallel rod axis to the covering coverslip. A single rod photoreceptor was centered in the imaging window and 8 bit images in gray scale were scanned. Images acquisition was performed using the LSM-510 software driving a confocal LSM-510 imaging system (Zeiss). At least five cells from two tadpoles were imaged and analyzed. Retinal tissues placed in frog Ringer’s solution were scanned by laser lines of 488 nm (for eGFP) and 543 nm (for mCherry). The scanning objective for both channels was a Plan-Neofluar 63×/1.4 oil lens (Zeiss). To minimize the bleeding through channels, emitted light from 488 nm excitation was filtered by a 500–535 nm, and those emitted by the excitation from the 543 nm laser, were filtered by a 655–710 band pass filter. The resolution of all scanned images was set to 0.04 × 0.04–0.08 × 0.08 μm in *xy* plane. (note: Based on Nyquist sampling theorem it is recommended to use two times of the diffraction limit, which for 488 nm is r = 209 nm and 0.1 μm provide the appropriate resolution without the risk of photobleaching [6]. However, we were able to resolve the mitochondrial membranes only at 0.04 × 0.04 μm in *xy* plan). At least five *z* scans from the central area of rod photoreceptor with a 0.5 μm interval were obtained. The LSM-510 software was set to correct for the *z-*plane while scanning dual channels. The pixel time, pinhole diameter, amplifier offset and amplifier gain were set to, 3 μs, 1.4 airy units, 0.1 and 1, respectively for all scans. Recombinant eGFP and mCherry at different concentrations can be scanned at the same settings used for retinal tissue imaging as described elsewhere [6].

8. *Image analysis for density measurement:*

Average pixel intensity were measured using AxioVision™ software version 4.8 (Zeiss), corrected for the background intensity. The averaged values of at least three *z* sections (each z sections may be corrected for photobleaching [19]) were used for density measurements. Deconvolution of images can be performed using theoretical mode in AxioVision 4.8 (Zeiss) or ImageJ. Accordingly 4–5 *z* planes needs to be deconvolved and used for resolving ultrastructures such as mitochondrial membrane [17]. We used recommended setting of AxioVision 4.8 (Zeiss). The optimal setting might vary based on the imaging goals.

### Method validation and results

*Examination of the retinal tissues and photoreceptors*

**Fig. 1:**
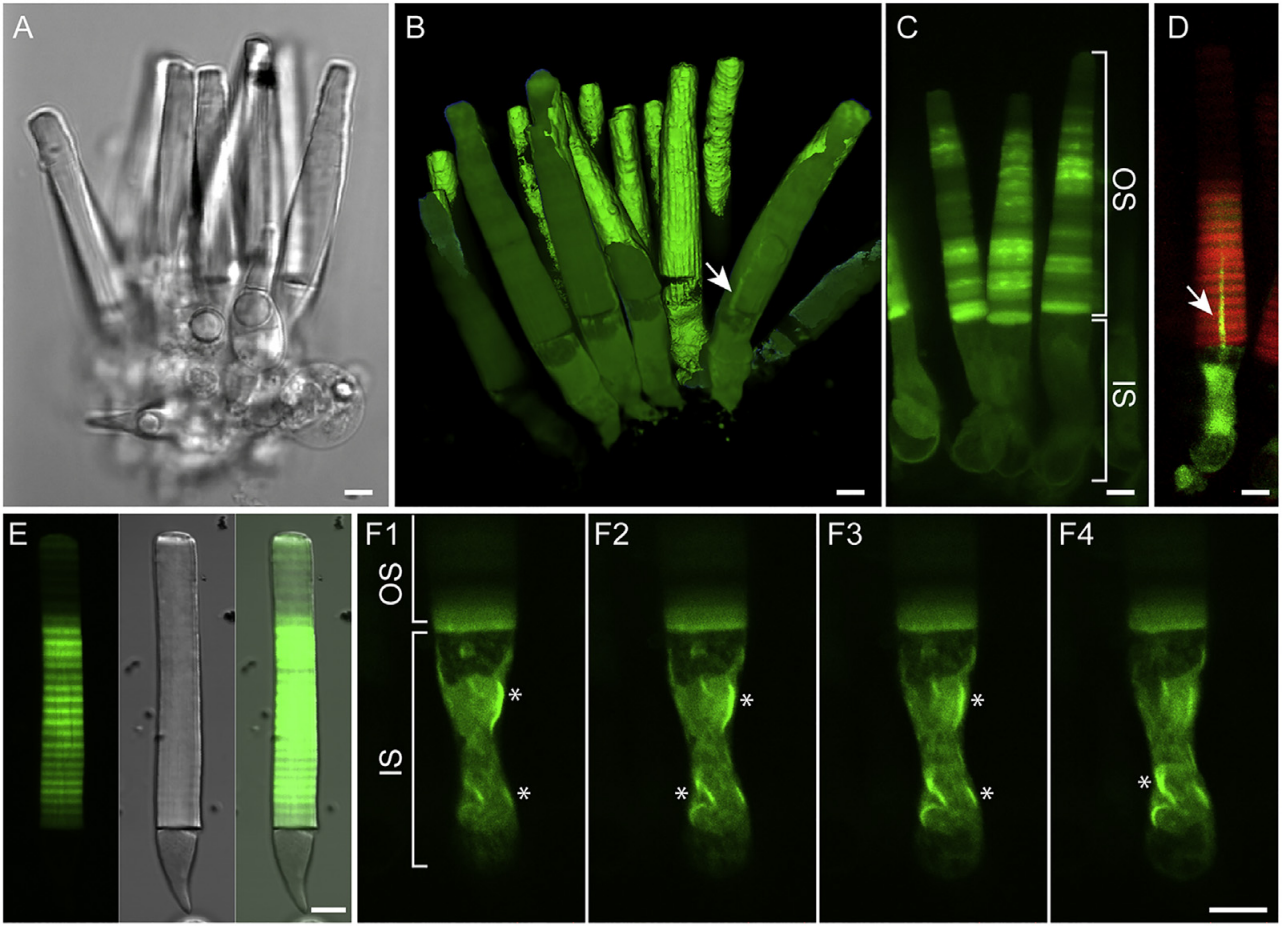
(A) A healthy live *Xenopus laevis* retinal tissues with healthy cells imaged with confocal microscope while bathed with frog ringer solution. (B) Live retinal tissue prepared from transgenic frogs expressing soluble eGFP. Using AxioVision 4.8 a surface was rendered on florescent cells and by rotating the x, y and z planes part of the surface was removed. The arrow shows the axoneme. (C) Live piece of retinal tissue prepared from transgenic frogs expressing an eGFP tagged mutant opsin (P23H [11]). The distribution of tagged mutant rhodopsin in both inner and outer segment can be seen clearly. Some fluorescent foci resembling aggregates are seen in the outer segment. (D) A single live photoreceptor expressing eGFP tagged arrestin and mCherry tagged rhodopsin (Rho-mCherry), simultaneously. The Rho-mCherry is localized to the outer segment and the eGFP tagged arrestin is localized to the inner segment mostly while it can also be detected in the axoneme (arrow). (E) A high-frequency fluorescent banding caused by light-dark cycle is seen in a rod photoreceptor expressing rhodopsin-eGFP. Dark lines along the rod long axis are generated by incisures which are invaginations of the disk membrane deprived of rhodopsin [23]. (F) The expression of an eGFP-tagged mutant rhodopsin (P23H) results in the aberrant expression of mutant protein in the inner segment and the formation of aggregates (*). OS: Outer segment IS: inner segment. Scale bar is 5 μm.

After the retinal tissue preparation one should scan the chamber and find 10–15 healthy, well-oriented and proximal retinal tissues (within 50 μm of coverslip). Retinal tissues might have a side-on or end-on orientation. The side-on orientation, where photoreceptor long axis is parallel to the coverslip, is the favorite orientation for the majority of applications. Healthy retinal tissues should not have many broken and damaged photoreceptors. A thin-cut retinal tissue with few cells provides the best setting for live imaging of photoreceptors and will result in less image-blurring (Fig. 1). In an optimal situation the depth of imaging is within 50 μm of coverslip. Photoreceptors within 10–20 μm of coverslip at both ends will generate the best images and are suitable for more accurate quantification of fluorescent tags.

*Image acquisition of fluorescently tagged proteins in rod photoreceptors*

Here we demonstrate a 3D reconstruction of live photoreceptors expressing soluble eGFP (Fig. 1, Movie) where single photoreceptors are observed along with photoreceptor ultrastructures, such as axoneme (arrow in Fig. 1B). Similarly, an eGFP tagged mutant rhodopsin expressed in rod photoreceptors demonstrates aberrant localization of the fusion protein in the inner segment (Fig. 1C). The expression of another mutant opsin (P23H) in rod photoreceptors demonstrates the aberrant localization of fusion proteins in the inner segment and the formation of dense fluorescent foci resembling protein aggregates [11] (Fig. 1F).

Other structures in photoreceptors or variation in the expression of transgenes caused by light/dark cycles can also be visualized with live imaging with high resolution. We demonstrated a high-frequency fluorescent banding pattern in photoreceptors expressing eGFP-rhodopsin caused by light-dark cycle (Fig. 1E). Alteration of the light-dark cycle to constant dark or light obliterated the reported fluorescent banding [17]. Live imaging of photoreceptors can be performed in photoreceptors expressing more than one transgene [11]. We generated transgenic tadpoles coexpressing eGFP tagged arrestin [6] and mCherry tagged rhodopsin (Fig. 1D).

**Fig. 2:**
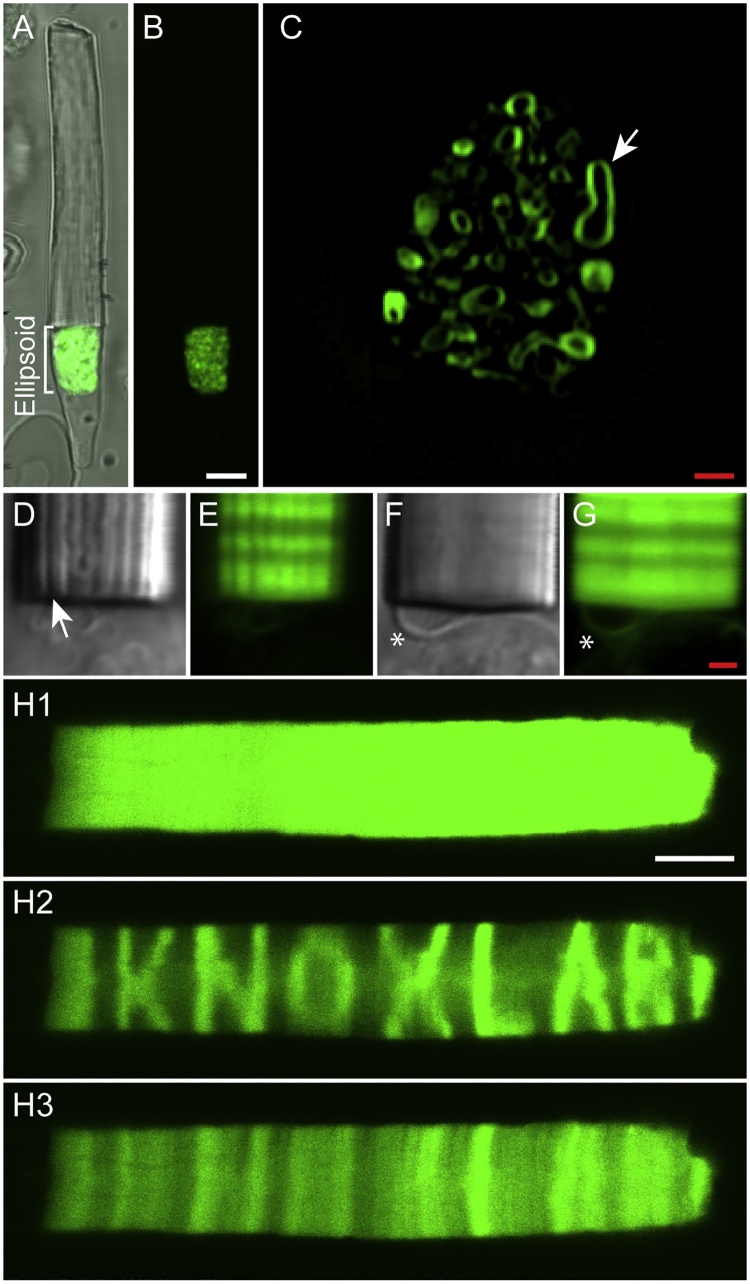
(A–C) A single rod photoreceptor expressing a tagged eGFP that is targeted to the mitochondrial membrane. Higher resolution of the photoreceptor ellipsoid followed by deconvolution demonstrates the distribution of the tagged eGFP in the mitochondrial membrane (arrow). (D & E) The depression in the outer membrane layer covering the outer segment is caused by incisures. This depression can be seen as a dark line along the rod axis with live imaging of freshly isolated photoreceptors (arrow). (F & G) The base of a rod photoreceptor expressing rhodopsin-eGFP. A pack of disks attached to the outer segment and inner segment boundary is seen that contains eGFP (*). (H) Photobleaching of a rod photoreceptor expressing double geranylated eGFP shows the outer segment before photobleaching (H1), right after photobleaching (H2) and 50 s after photobleaching (H3). The double geranylated eGFP is free to diffuse laterally (within the disks and along the rod short axis) and therefore it demonstrates the recovery after photobleaching along the disk axis. There is minimal recovery along the rod long axis, on the other hand, due to trivial movement of double geranylated eGFP along the rod axis.

Other organelle structures in photoreceptors, such as mitochondria can be visualized in high resolution. We expressed a tagged eGFP targeted to the membrane of mitochondria and were able to resolve the mitochondrial membrane by deconvolving the images obtained from live imaging of photoreceptors (Fig. 2). Unique features in photoreceptors such as, depressions in the outer segment membrane caused by incisures were observed with live imaging. We also recorded fine structures at the base of outer segment possibly resembling a packet of disks made during daily disk synthesis (Fig. 2).

*Performing FRAP on live rods*

The capability of performing FRAP is an advantage limited to live imaging and is not possible in fixed tissues. We studied the lateral and axial diffusion of different fluorescently tagged proteins expressed in photoreceptors [11]. As an example we performed FRAP analysis in photoreceptors expressing double geranylated eGFP (eGFP-dGrygry). A single photoreceptor expressing eGFP-dGryGry was bleached and demonstrated substantial recovery after 50 s of time-lapsed live imaging (Fig. 2H).

### Summary

We demonstrated several examples of fluorescently tagged proteins expressed in rod photoreceptors that provided valuable information about dynamics and transport of proteins in rod photoreceptors [2,11,17,20]. We also gave a summary of different steps leading to a successful live imaging of rod photoreceptors. Live imaging of retinal tissues has been described previously by other investigators who were the pioneers [6]. However, the complexity of the technique cannot be explained by words. Therefore, this manuscript is complemented by a video (see Supplemental data) to show the way retinal tissue is prepared and imaged to allow other investigators to employ this valuable technique. Live imaging of retinal tissues is a suitable method to study the transport, localization, interaction and dynamics of different proteins in highly specialized photoreceptors. This approach can be utilized to learn more about the pathogenesis of eye diseases and potential therapies [8,17,[20], [21], [22]]. These types of studies are not suited for fixed tissue imaging.
